# Occupational Performance Coaching with Parents to Promote Community Participation and Quality of Life of Young Children with Developmental Disabilities: A Feasibility Evaluation in Hong Kong

**DOI:** 10.3390/ijerph17217993

**Published:** 2020-10-30

**Authors:** Chi-Wen Chien, Yuen Yi Cynthia Lai, Chung-Ying Lin, Fiona Graham

**Affiliations:** 1 Department of Rehabilitation Sciences, The Hong Kong Polytechnic University, Hung Hom, Kowloon, Hong Kong (SAR), China; cynthia.yy.lai@polyu.edu.hk (Y.Y.C.L.); cylin36933@gmail.com (C.-Y.L.); 2 Rehabilitation Teaching and Research Unit, University of Otago, Wellington South 6242, New Zealand; fi.graham@otago.ac.nz

**Keywords:** occupational performance coaching, community participation, health-related quality of life, Hong Kong, preschool-aged children, developmental disability

## Abstract

Participation in community activities contributes to child development and health-related quality of life (HRQOL), but restricted participation has been reported in children with disabilities. Occupational performance coaching (OPC) is an intervention that targets participatory goals in child performance through coaching parents, with evidence of effectiveness for pediatric populations. Little is known about the feasibility of OPC in Hong Kong, or its effect on children’s community participation and HRQOL. A mixed-methods case study design was applied to explore Hong Kong parents’ experience of OPC in relation to goal achievement, community participation, and HRQOL change in children. Four parents of young children with developmental disabilities (aged five to six years) received OPC for three to eight sessions within one to three months. Quantitative pre- and post-intervention data were analyzed descriptively. Semi-structured interviews with parents were conducted at post-intervention, and analyzed using content analysis. Results showed a trend of improvement in goal performance, child involvement in community activities, and specific aspects of HRQOL among most participants. Parents perceived undertaking OPC positively, described gaining insights and skills, and felt supported. The findings suggest that OPC warrants further investigation for use in Hong Kong, to promote children’s community participation and quality of life.

## 1. Introduction

The opportunity to participate and be involved in community activities is necessary for the optimal physical, emotional, and psychological development of children [1,2,3]. Community participation allows children to make friends, learn skills, and develop independence and a sense of belonging. Yet, children with developmental disabilities (DD) as young as five, participate less frequently, and are less involved in community activities, compared to children with typical development [4,5,6]. While DD includes a heterogeneous group of impairments [7,8], lower community participation may, in itself, impede the development of children with DD [9,10], adversely affecting their health and quality of life [11,12]. Research that focuses on improving community participation for young children with DD is urgently needed [13,14].

A recent systematic review of community participation interventions in children and adolescents with DD [15], found 13 interventions that improved friendships, recreational participation, and quality of life. Few interventions were identified that were designed for and applied to children younger than six years. Current models providing early intervention services focus predominantly on body impairment, or incapacity to execute daily [16]. However, evidence indicates that these types of interventions do not necessarily contribute to improve children’s participation in real-life, practical situations [17,18]. Instead, as changes in participation are considered multifactorial [2,19,20], approaches should be individually-tailored, family-centered, and ecologically-oriented.

Coaching has recently been highlighted as an evidence-based intervention that engages parents of young children in early intervention and pediatric rehabilitation [16,21,22]. Coaching is defined as partnering with clients in a thought-provoking and creative process that maximizes their personal and professional potential [23]. In pediatric rehabilitation, this takes place in family settings, by collaboratively working with parents on individualized participatory goals, identifying parents-directed solutions, and building their capacity to implement practical strategies [24].

Occupational performance coaching (OPC) [25] is one of several coaching interventions that are applicable to children with DD. OPC facilitates children’s occupational performance and participation through coaching parents to implement change in the context of children’s life situations. Key techniques in OPC include mindful listening, empathy, focusing on parents’ priorities, collaborative performance analysis, and sharing knowledge. These techniques are used to heighten parents’ engagement in the action-reflection process [25,26,27]. OPC is non-directive in that parents are not advised, instructed, or trained in any action or method. Instead, using goal-specific, open-ended questions, therapists guide parents to identify highly individualized and practical strategies to improve children’s participation. As such, OPC takes an enablement-focused, family-centered, and ecologically-oriented approach to address participation difficulties faced by children with DD and their families [25].

Emerging evidence that supports the effectiveness of OPC includes case studies [28,29,30], time-series [31], and randomized controlled trial designs [32]. The effects of OPC on parents’ wellbeing, including self-competence, have also been demonstrated [28,31,32]. However, the extent to which OPC leads to changes in community participation is unclear, given individualized measures of personally-identified participation goals were used in all of previous studies, with no subgroup analysis of community participation effects. Furthermore, few studies investigated whether OPC could improve children’s quality of life or parents’ emotional states. To date, existing evidence for OPC has been established in Germany [30], Australia [28], Canada [29], and Iran [32], but more research is needed to test its feasibility when applied to parents with other cultural backgrounds.

Hong Kong has a culture influenced strongly by Chinese collectivism [33,34], and there seems to be a lot of stigma and shame surrounding children with disabilities and their families [35,36]. Consequently, this could lead parents to withdraw themselves and their children from social situations [37]. Indeed, young children with DD in Hong Kong have been reported to participate less in community activities [6,38], compared to those in other countries [4], and this decreased participation appears to correlate significantly to their parents’ parental stress [6,38]. These issues highlight the need for an effective approach that supports parents and their young children with DD and promotes community participation.

The primary aim of this study was to investigate the feasibility of OPC in Hong Kong, with parents of young children (aged < 6 years) with DD, to promote children’s community participation and health-related quality of life (HRQOL). Moreover, we also aimed to explore parents’ experience of OPC, its effect on their emotions, and their perception of autonomy support from OPC (compared to conventional early intervention services). Specifically, the research questions were: (1) Can OPC lead to improvement in community participation and HRQOL of young children with DD? (2) Can OPC lead to improvement in parents’ self-competence, emotional states, and perceived autonomy support? and (3) What are parents’ experiences of being coached with OPC?

## 2. Materials and Methods

### 2.1. Study Design

A mixed-methods case study design was used to examine the feasibility of applying OPC to Hong Kong parents of young children with DD. Both quantitative and qualitative methods were used to collect and analyze case study data. Quantitatively, pre-post intervention measures were used to describe children’s community participation and HRQOL. Parents’ goal performance and satisfaction, self-competence, emotional states, and perceived autonomy support were also measured quantitatively. The qualitative approach utilized a semi-structured interview to explore parents’ experience of OPC after the intervention. Ethical approval for the study was granted by the Human Subjects Ethics Sub-committee at The Hong Kong Polytechnic University (number: HSEARS20190114005).

### 2.2. Participants

Parent-child dyads were recruited, via convenience sampling, from three local non-governmental organizations that provided early intervention services to preschool-aged children with DD. Inclusion criteria were: (1) the child had been diagnosed with developmental delay, autism spectrum disorder, intellectual disability, or attention deficit/hyperactivity disorder, by local multidisciplinary child assessment centers; (2) the child was aged between two and five years (inclusive); (3) parents were able to read Chinese; and (4) parent(s) were the main caregiver(s) of the eligible child. Children with comorbidities of specific physical impairment (e.g., cerebral palsy or amputation), blindness, or deafness, were excluded from the study. This is because children with physical/visual/hearing constraints might need more complex environmental modifications or provision of assisting devices for participation in community activities, which were tentatively excluded from the present study. Written consent was obtained from the parents prior to research participation.

### 2.3. Instruments

#### 2.3.1. Parent-Identified Community Participation Priorities

The Chinese version of the Canadian Occupational Performance Measure (COPM) [39] was used to measure parents’ perceptions of children’s community participation. The COPM identifies individualized problems in participation in occupations through a semi-structured interview. A two-point or larger difference in COPM scores between pre-post interventions is considered clinically important [39]. The COPM has adequate test-retest reliability [40] and internal consistency (Cronbach’s alpha = 0.73–0.88) [41].

Parents were asked to identify goals related to their child’s participation, and to rate the child’s performance and their satisfaction with current performance on a 10-point Likert scale (1 = not good/satisfied at all, and 10 = optimal performance/satisfaction). Consistent with COPM and OPC, parents were invited to identify goals related to any life areas. In addition to COPM protocol, parents were invited to identify at least one goal for their child’s community participation.

#### 2.3.2. Parent-Reported Community Participation in Children

The community section of the Young Children’s Participation and Environment Measure (YC-PEM) [4], a caregiver-report questionnaire, was used to evaluate the extent of children’s participation in various community activities. Parents were asked to complete 10 items regarding community activities such as outings, class and group activities, community events, and recreational/leisure activities. For each item, parents evaluated three dimensions of child participation: (a) the frequency of participation, using an eight-point Likert scale (never = 0, and once or more each day = 7); (b) the degree of involvement, using a five-point Likert scale (not very involved = 1, and very involved = 5); and (c) whether the caregivers want a change in their child’s participation (yes or no, if yes, specify the type(s) of desired change). Three types of participation summary scores (frequency, involvement, and desire for change) can thus be generated, and the score calculation was detailed in Khetani et al.’s study [4]. We analyzed the frequency and involvement dimensions because they are two important aspects representing children’s participation patterns [20].

The community section of the YC-PEM has adequate test-retest reliability (interclass correlation coefficients (ICC) = 0.84–89) [6]. Minimal detectable change (MDC) values of 0.7 points were also established for both the frequency and involvement scales [6]. Internal consistency of the YC-PEM was acceptable for the frequency (Cronbach’s alpha = 0.64–0.68) and involvement (Cronbach’s alpha = 0.77–0.96) scales in its community section [4,6].

#### 2.3.3. Parent-Reported HRQOL in Children

The parent-report version of the Kiddy-KINDL questionnaire was used to measure HRQOL in children aged three to six years. The Kiddy-KINDL comprises 24 items that assess parents’ perceptions of their child’s HRQOL across six dimensions: physical wellbeing, emotional wellbeing, self-esteem, family, social contacts, and school functioning. The recall period was pre-set as the last month in this study, and each item is rated using a five-point Likert scale (0 = never, and 4 = all the time). Item scores were summed up to indicate dimension scores, and these were summed up to indicate an overall score. Raw dimension and overall scores were subsequently transformed into a scale of 0–100 to facilitate interpretation [42]. The Kiddy-KINDL has demonstrated acceptable internal consistency (Cronbach’s alpha = 0.70–0.89) [43,44].

#### 2.3.4. Parenting Self-Competence in Parents

The Parenting Sense of Competence Scale (PSOC) [45] comprises 16 items, and was used to obtain parents’ perception of their parenting role in the two dimensions of efficacy and satisfaction. Parents were asked to rate each item on a six-point Likert scale (6 = strongly disagree, and 1 = strongly agree). Total scores were generated by summing all items in each subscale (after reversing the negatively worded items). The PSOC has demonstrated good test-retest reliability (ICCs = 0.82–85) and internal consistency (Cronbach’s alpha = 0.77–0.80) [45].

#### 2.3.5. Self-Reported Emotional States in Parents

The Depression, Anxiety and Stress Scale-21 (DASS-21) [46] was used to measure parents’ negative emotional states of depression, anxiety, and stress. It is a self-report questionnaire and includes 21 items (7 items for each subscale of depression, anxiety, and stress). Each item is rated on a four-point Likert scale (0 = did not apply to me at all, and 3 = applied to me very much or most of time). Total scores were generated by summing all items in each subscale and multiplying by two. Good internal consistency (Cronbach’s alpha = 0.77–0.87) of the DASS-21 has been reported [46].

#### 2.3.6. Parents’ Perceived Autonomy Support from Health Care Practitioners

The Health Care Climate Questionnaire (HCCQ) [47] was used to measure the degree to which parents perceived how their health care practitioners encouraged their autonomy. In this study, the term “health care practitioners” was changed to their child’s occupational therapists at pre-intervention, and to OPC coach at post-intervention for comparison. Parents were asked to respond to 15 items regarding their relationship with the occupational therapist (OPC coach), on a seven-point Likert scale (1 = strongly disagree, and 7 = strongly agree). Mean of the 15 items was calculated to create the HCCQ index. Good internal consistency (Cronbach’s alpha = 0.95) of the HCCQ has been reported [47,48].

#### 2.3.7. Demographic Information

A parent-reported questionnaire was designed to collect demographic information such as child age and gender, family structure, family income, and both parents’ age, occupation, and education. During telephone screening, parents were also asked to report the type(s) of clinical diagnosis their child had obtained from the reports of child assessment centers, and rate the severity of their child’s disability as a whole, using a four-point Likert scale (1 = very mild, and 4 = severe). In addition, participants’ names were collected but replaced by the numbers in this study, allowing for confidentiality when reported.

#### 2.3.8. Parents’ Experience of OPC Intervention

A semi-structured guide was developed to elicit parents’ experience of OPC. This interview guide included a list of open-ended questions, as well as related probes, allowing direct questions with flexibility when pertinent information emerged during the interview. We designed the guide to explore multiple aspects of parents’ interview experience regarding their perceptions, satisfaction, perceived effects, process, and suggestions on OPC intervention. Appendix A details the guiding questions used in the interview.

### 2.4. Procedure

Research invitations were distributed to eligible participants through occupational therapists working in non-governmental rehabilitation services. Parents who were interested in participating contacted a research assistant, and were screened for eligibility during a telephone conversation. Following signed consent and enrolment in the study, pre-intervention measures (the YC-PEM, Kiddy-KINDL, PSOC, DASS-21, HCCQ and demographic questionnaire) were posted to parents, two weeks before intervention. During the goal setting session, the COPM was administered, by the first author (Chi-Wen Chien), at a location of the parents’ choice.

Subsequently, parents attended a maximum of eight weekly sessions of OPC (each for one hour at most). OPC sessions were delivered through several modes, including face-to-face at a location of the parents’ choice, or through Zoom video communications (Zoom, San Jose, CA, USA). Consistent with OPC guidance [25], children’s attendance at the coaching sessions was at the parents’ discretion. During the study period, either parents, their child, or both, continued pre-existing service engagement.

One week after the completion of the OPC sessions, the parents repeated all outcome measures. The COPM was completed with the research assistant, who was blind to the treatment content. The research assistant also conducted the post-intervention interview of the parents’ experience of OPC. All interviews lasted 20–40 min, and were conducted via Zoom and audio-recorded. At the two month follow-up, all measures except for the HCCQ were repeated a third time.

### 2.5. OPC Intervention

OPC was delivered by the first author (Chi-Wen Chien), who is an occupational therapist and researcher. He attended a three-day training workshop conducted by the last author (Fiona Graham), the OPC developer. Prior to the study, he practiced with five parents of children with and without disabilities (achieving the fidelity ratings at an average of 84.4%), and received ongoing guidance from the OPC developer to ensure his fidelity of OPC.

The OPC sessions involved techniques comprising the three enabling domains described by Graham et al. [25]: connect, structure, and share. Connect refers to building parents’ trust in the therapist, by using verbal and nonverbal strategies such as mindful listening, empathizing, and partnering, to help parents shift from an emotional (reactive) to a solution-focused (proactive) orientation. Structure alludes to building parents’ competence, by guiding them through a problem-solving framework of setting goals, exploring options, planning action, carrying out plans, checking performance, and generalizing. Share refers to optimizing parents’ autonomy, by emphasizing and building on parents existing knowledge, skills, and resources.

In the first OPC session, parent(s) and the therapist identified one goal that was currently important to the parent(s), regardless of the performance and satisfaction ratings provided in the COPM. The therapist engaged parents in collaborative performance analysis of that goal, by following the four steps to: (a) identify parents’ perception of what currently happened, (b) identify what parents would like to happen, (c) explore barriers and bridges to the desired performance, and (d) identify their needs for taking actions to achieve goals. Each session ended with clarification of the action plan for the following week. In subsequent sessions, parents were prompted to review the usefulness of planned actions to achieve goals. When strategies were useful, the therapist guided parents to generalize their application to other aspects of life. Unsuccessful strategies became discussion points to review goals, knowledge, and alternative ways of engaging in goal activities.

### 2.6. Data Analysis

Case descriptions were developed to characterize participating children and parents, specify parents’ goals, and describe the progress of OPC sessions. Quantitative data were next analyzed using descriptive statistics such as mean, standard deviation, and proportion, and were reported in table forms. No inference statistical analysis was performed because of the nature of the descriptive case study design with a small sample size.

For qualitative data, audio-recorded interviews were transcribed verbatim. Conventional content analysis was used to analyze the interview data [49,50]. Specifically, the first author (Chi-Wen Chien) initially read the transcripts to obtain a general sense of the content. The analysis of manifest content was followed by open coding process independently done by the two coders. In the process, they generated the codes inductively, and read transcripts again to refine and condense codes into extended meaning units, before placing similar codes together where they fitted under an emerging category or sub-category. Once preliminary categories and sub-categories, if needed, were generated, the two coders met and reviewed the coded data to determine if each category/sub-category formed apparently coherent patterns with sufficient supporting data. Discrepancy was discussed and the final list of categories and sub-categories was determined through consensus among the coders.

## 3. Results

### 3.1. Case Descriptions

Initially, six parents participated in this study, completed pre-intervention questionnaires, and attended the goal setting sessions (see Table 1). Cases consisted of five boys and one girl, ranging from 4–5.5 years of age. Most of the children had been diagnosed with either DD, autism, or both, and the parent-perceived severity of child disability was reported as mild or moderate.

Each parent identified five to eight goals (mean = 6.7; SD = 1.0) and, of those goals, between one and three (mean = 1.8; SD = 0.8) were related specifically to the child’s community participation (see Table 2 for details). Coached participants included one pair of parents, four mothers, and one father. After the first OPC session, two mothers withdrew from the study due to child illness (*n* = 1), and preference for an expert-directed approach (*n* = 1). The remaining four participants are included in the analyses. Parents received three to eight coaching sessions (mean = 5.8 and SD = 2.1), across 5–11 weeks (mean = 8.3; SD = 2.8), dependent on goal achievement. Detailed information on coaching sessions and delivery modes is provided in Table 1. Appendix B provides narrative descriptions of OPC intervention processes and goal achievement in each session.

### 3.2. Quantitative Results

For parent-identified goals as measured by the COPM, the differences between pre- and post-intervention were greater than or equal to two points in the performance and satisfaction of 19 (73.1%) of 26 goals, and for 5 (71.4%) of 7 goals specific to community participation (see Table 2). Goal performance and satisfaction decreased slightly at two months follow up, but were maintained beyond clinically important levels in terms of the average among the four parents (see Table 3).

For children’s community participation, as measured by the YC-PEM, there was a trend of positive changes in all four children’s involvement. The average change scores were 0.6 and 0.4 between pre- and post-intervention, and between pre-intervention and follow-up, respectively. However, the magnitude of the average change scores did not exceed the MDC value of 0.7 points of the YC-PEM [6]. On the contrary, there was a trend of a small decrease in the participation frequency scores of half the children, between pre- and post-intervention. The magnitude of the decrease in the children’s participation frequency between post-intervention and follow-up and between pre-intervention and follow-up (see Table 3) was larger than the MDC value of 0.7 points, indicating a true decrease beyond the random measurement error.

Table 4 shows the results of outcome measures in relation to children’s HRQOL and parent-related outcome. For HRQOL as measured by the Kiddy-KINDL, two to four children were reported by their parents as having a tendency to experience a positive increase in physical wellbeing (mean change = 12.50), family (mean change = 4.69), and school functioning (mean change = 4.69) after OPC intervention, compared to their baseline status. However, except for self-esteem, all aspects of HRQOL tended to decrease negatively between post-intervention and follow-up. By considering the entire study period between pre-intervention and follow-up, only the physical wellbeing had a positive increasing trend (mean = 7.81) in all the four children.

A similar pattern was observed in the parents’ emotional states and parenting competence. That is, the parents’ emotional problems, especially depressive symptoms as measured by the DASS-21, tended to improve after OPC intervention, but deteriorate at follow-up when compared to pre- or post-intervention (see Table 4). For parenting competence as measured by the PSOC, the change in parents’ satisfaction tended to increase at post-intervention, but decrease at follow-up. One exception was the parenting efficacy which tended to improve gradually at both post-intervention and follow-up period (mean change = 1.25 and 0.75, respectively). In addition, there was a trend that all four parents reported higher HCCQ scores for the OPC therapist’s autonomy-supportive behaviors, in comparison with their child’s occupational therapist (mean difference = 0.73).

### 3.3. Qualitative Results

Four major categories (with 12 sub-categories in total) in relation to the parents’ experience of OPC intervention were identified from the coding of their post-intervention interviews. These included: (1) increased insight and learning, (2) experiencing changes in their child, (3) positive coach-parent relationship, and (4) factors affecting coaching experience and suggestions. Table 5 shows a summary of the four categories and 12 sub-categories, and illustrative quotations under each sub-category are provided in Appendix C.

#### 3.3.1. Increased Insight and Learning

All parents considered their coaching experience to have contributed to an increased insight of their and their child’s needs. For example, the mother of Case 3 realized that her lack of time-management skills hindered the implementation of effective strategies to her child’s morning routine. Shifting her focus to her own time management led to goal progress. The other three parents reported an increased understanding of their child’s emotions or learning styles, which allowed them to explore or adjust strategies to meet the child’s needs. Most parents reported that access to strategies, skills, or thinking models during OPC, enabled them to facilitate their child’s activity participation.

#### 3.3.2. Experiencing Changes in Their Child

Three of the four parents reported their children participated more in home activities, following the OPC, for example, children were more engaged in doing homework or playing with siblings at home. The parents also observed changes in their child’s emotions or confidence at home, at school, or in the community. However, when asked about whether OPC had helped with their child’s participation in community activities, no to little improvement was reported.

#### 3.3.3. Positive Coach-Parent Relationship

Positive partnership between the therapist and parents was a major category, which contributed to parents’ perceptions of how OPC had helped to facilitate their child’s participation. Those parents felt supported by the therapist to guide the solution-focused thinking process, or felt encouraged to focus on goal achievement, with constant experimentation of suitable strategies. The parents also felt understood and accepted by the therapist.

#### 3.3.4. Factors Affecting Coaching Experience and Suggestions

The parents expressed a consistently high level of satisfaction regarding the coaching process. For example, the mother of Case 5 said *“The parent-coaching process is very good. The 1-h meeting drove us to be very focused.”* The most common word to describe their perceptions of the OPC process was *“satisfied”*. However, the parents commented that their experience of coaching had been compromised either by the social unrest, seasonal holidays, or both, particularly when their child was unable to go to school as usual. Two parents (Cases 5 and 6) also preferred the face-to-face coaching mode, but the father of Case 6, who had had coaching in his car due to it being the quietest option, was displeased with the lack of formality. On the other hand, the parents of Cases 1 and 3 enjoyed the advantage of having internet-based coaching.

In addition, the parents provided several suggestions regarding the application of OPC in Hong Kong, for example, an increase in the total number of coaching sessions but a decrease in their frequency, would give the parents more time to try the planned strategies, or see the improvement, especially during seasonal holidays. Access to a resource book, email/mobile message reminders, or parents’ education before or during OPC, were some of the other suggestions.

## 4. Discussion

The case studies evaluated the feasibility of OPC with Hong Kong parents, to promote community participation and HRQOL of their young children with DD. Overall, quantitative results indicated clinically meaningful gains in the performance and satisfaction of parents’ identified goals regarding children’s community participation after OPC intervention. A trend for the post-intervention gains were also revealed in children’s participation involvement in community activities, although only a relatively small improvement in the children’s HRQOL was observed after OPC. Most parents tended to experience an increase in their parenting self-competence and perceived autonomy support. This concurred with qualitative findings that parents engaged in the OPC process positively, gained insights about their child and themselves, learnt new skills/mindsets, and felt supported. The parents’ positive engagement and learning in the OPC process might help them in facilitating children’s participation and emotions. Additionally, parents provided several suggestions on the OPC process in Hong Kong, which warrant consideration for future studies.

Coaching has been increasingly used as the core approach in several interventions that have been found to promote children’s community participation [51,52,53]. Similarly, our case studies also support the use of OPC to achieve parents’ aspirations regarding children’s participation in community activities. Nevertheless, in this study, not all community-related goals were addressed during OPC sessions. According to post-intervention interviews, parents perceived change in their child’s participation mostly at home. We thus think that the increase in children’s community participation may have resulted from the generalization effect of OPC, as reported in previous studies [28,31,54]. This is because, during OPC, parents’ generalization of successful strategies is encouraged by explicitly asking them about other areas to which the strategies might apply [25]. For example, the mother of Case 1 reported that her child became cooperative in his extracurricular piano lessons, after she shared the strategy with the teacher that had helped motivating her child to do homework. Parents also reported a range of enhancements to their capacity, and showed an increase in their parenting efficacy and autonomy. These findings reflect the possibility of changing parents’ mindsets or behaviors, empowering them to be active in supporting their child’s involvement in community activities.

Contrary to the trend of the increased involvement in community activities after OPC, no increase in the frequency of community activities among children was observed from the results of the YC-PEM. The different nature of community activities may be one possible reason for the finding. For example, some community activities occur regularly (e.g., weekly extracurricular lessons), whereas others are held on specific occasions (e.g., summer overnight trips, parades). Furthermore, preschool children, as they are young, tend to have regular daily routines [55], making little room for them to take part in community activities more often. Maul and Singer [56] found that some types of community activities (e.g., going to crowded places or shopping malls) were avoided by parents of young children with disabilities. Additionally, during 2019–2020, protests against the extradition bill took place over the weekends in Hong Kong [57], when the case studies were carried out. This also coincided with the outbreak of Coronavirus Disease 2019 (COVID-19) in early 2020, which rendered children and people to self-isolate and, in turn, might affect the primary outcome of the present study (i.e., frequency of community activities).

Improved parent-identified goal performance using OPC appeared to be translated into increased HRQOL of children, even though the increase was small, domain-specific, and of unclear clinical significance. We found a trend that some children had higher physical wellbeing and family and school functioning after OPC, perhaps because their parents developed increased insights about the child, and learnt handling skills/strategies. Those parents might know how to arrange activities and optimize their child’s vitality, manage conflict between the child and themselves, and enable the child to complete homework. On the contrary, no improvement, or even a decreasing trend for the children’s psychosocial aspects of HRQOL (i.e., emotional wellbeing, self-esteem, and social contacts with friends) was noted. This could be explained by the systematic impact of social unrest, as mentioned above, causing the families to stay at home and feel unhappy [58,59]. The trend of decreased emotional wellbeing and self-esteem, however, was somewhat contradictory to the findings of some parents’ post-intervention interviews, where OPC was indicated to benefit the child’s emotions and confidence. We speculated that such improvement could be specific to certain contexts, which may not be reflected by comprehensive HRQOL measures. Given that the finding is preliminary at the case-study level, continued studies are warranted to clarify the effect of OPC on children’s HRQOL.

Consistent with previous findings of OPC [31,32], the parents in this study tended to show improvements in their sense of efficacy in parenting. We also found that those parents felt supported and understood by the OPC therapist, and perceived the therapist as more supportive of their autonomy, compared to their child’s occupational therapist. This might be because traditional early intervention tends to focus directly on children, whereas coaching is a highly collaborative approach highlighting close partnership with the family [22,60]. In OPC, parents can identify goals meaningful to them, create their own strategies, and plan with the coach when to implement such strategies in practice. Furthermore, some parents in this study tended to report a small reduction of their stress, anxiety, and depression at post-intervention. This suggests that the tendency for their improved emotional wellbeing may be related to that for either their increased self-efficacy, autonomy support, or both, gained from OPC. This is consistent with the findings of Dunn et al.’s study [61] which used similar coaching approaches, and parents reported increased parental efficiency but decreased distress. Thus, coaching may lead to improvements, in not only child-related, but also parent-related outcomes, including self-efficacy, autonomy, or even emotional states [60].

The parents in this study generally expressed satisfaction with the OPC process but, inevitably, their experience was compromised by the social unrest, which impacted on the delivery mode and locations of intervention. From post-intervention interviews, we noticed that the parents of Cases 1 and 3 enjoyed having internet-coaching in their homes, while the other two parents favored face-to-face modalities, either at home or in a formal location. This suggests that, regardless of the coaching mode used, parents seem to prefer interventions that focus on the home environment, as this could be more useful to their child. As the comparative influence of remote versus face-to-face use of OPC is not yet fully understood [28], it might be preferable to use one or the other in a consistent manner, while tending to each participant’s preferences and needs.

All parents suggested the necessity of having either more sessions, time between the sessions, or both. These suggestions were expected, as three of the four participants had merely three or six sessions, owing to the impact of COVID-19. There were also unforeseen variations in family schedules during seasonal holidays, or school suspension caused by the social unrest. Parents thus needed to cope with the variations immediately, and were unable to try out the planned action agreed upon during each OPC session. To accommodate such situations, and allow for more time to implement the plan, we decided to reduce the frequency of the sessions from weekly to weekly/fortnightly in future interventions. The total number of eight sessions, however, will be kept the same, given that the coaching frequency has been reduced, and the entire coaching period is lengthened. We think that the parents’ additional suggestions (e.g., providing handbooks, reminders, or parental education) are more relevant to parent’s training, where therapists tend to instruct parents and demonstrate how to apply strategies in a straightforward manner. According to Akhbari Ziegler and Hadders-Algra [62], these are unlikely to fit into approaches like OPC, where the focus is on empowering and supporting parents in the process of decision making regarding strategies specific to their child’s participation in daily life activities.

Limitations of this study include the small number of case studies, the inclusion of all boys as the child participants and, especially, the influence of the social unrest and COVID-19 on study progress and outcome. For example, the parents attributed the lack of change in their child’s community participation to the fact that they had not gone out often between June and December 2019, when the social unrest was persistent. Furthermore, the fact that all participating children continued receiving their usual early intervention services during the study period might have acted as a confounding factor. Future studies, with larger samples of a balanced gender proportion, and using a randomized controlled trial design, with children assigned to either the OPC and usual care group, or usual care only, are warranted to evaluate additional contribution of OPC, and to confirm the findings of this study.

## 5. Conclusions

This study provides preliminary support for the use of OPC in parents of young children with DD in Hong Kong. We found a trend that OPC may have a positive effect both on children’s involvement in community activities and on specific aspects of quality of life. OPC can also assist parents in developing insight, skills, autonomy, and self-efficacy which, in turn, may benefit their emotional state. While satisfaction with OPC was high among the parents, some suggestions were useful to adjust the intervention to fit with local needs. These findings could help inform further planning of either a pilot, feasibility randomized controlled trial, or both, to establish evidence supporting the effectiveness of OPC when being applied in Hong Kong.

## Figures and Tables

**Table 1:** Details of demographic characteristics of participants and the OPC sessions delivered.

Characteristics	Case 1	Case 2	Case 3	Case 4	Case 5	Case 6
Age (years)	5.25	4.00	5.50	5.25	5.33	5.25
Gender	Boy	Boy	Boy	Girl	Boy	Boy
Diagnosis	Autism and DD	Autism	Autism and DD	DD	DD and dyslexia	Autism and DD
Parent-reported severity of disability	Mild	Moderate	Moderate	Moderate	Mild	Mild
Father/mother’s age (years)	50/40	45/43	33/36	38/37	44/43	45/32
Father/mother’s educational qualification	Bachelor/Bachelor	Form 5/Form 5	Bachelor/Bachelor	Postgraduate/Bachelor	Postgraduate/Postgraduate	A-level/Bachelor
Parent(s) being coached	Father and mother	Mother *	Mother	Mother *	Mother	Father ^†^
Number of coaching sessions received	6 ^‡^	1	8	1	6 ^‡^	3 ^‡^
Number of weeks	10	1	11	1	7	5
Delivery mode	Internet	Face-to-face	Internet	Internet	Face-to-face	Face-to-face

* Two parents withdrew from the study after attending the first session. ^†^ The mother joined the second coaching session with the father once. ^‡^ Coaching was terminated earlier owing to the outbreak of COVID-19. The parent(s) received face-to-face coaching in the first session but chose internet-based coaching for the remaining sessions. Abbreviation: DD, developmental delay.

**Table 2:** COPM scores for parent-identified goals for their children and themselves.

		Performance			Satisfaction		
	Goals	Pre	Post	FU	Pre	Post	FU
Case 1	Demonstrates stable emotion when talking to the parents or his old brother	5	7	8	6	7	8
	*Completes homework with concentration at home*	6	6	8	6	7	8
	Participates in school activities with concentration and cooperation	6	8	7	6	8	7
	Shows friendly and good interaction with classmates at school	5	7	7	5	7	7
	* *Engages in and keeps focused on the activities during the group’s interest classes*	5	7	8	6	8	8
	* Shows kindness and does not affect other children outside the home	5	6	7	6	7	7
	*Parents learn about the child’s emotions and know to deal with his emotional changes*	3	7	8	4	7	8
Case 2	Eats the dinner at home on his own by sitting on his chair and has more attempts to try different kinds of food	4	-	-	6	-	-
	Feels acceptable when having haircut at home or at hair salon	5	-	-	4	-	-
	* Eats the meals outside home with more concentration and not watching iPhone or iPad all the time	3	-	-	3	-	-
	* Feels more comfortable when taking public transportation (e.g., MTR, bus, or taxi) for outings	4	-	-	5	-	-
	Wears different clothes and shoes before going outside	4	-	-	6	-	-
	Completes the homework at home by sitting well on the chair	5	-	-	4	-	-
	* Parent finds suitable ways/approaches/strategies to bring the child outside when taking public/private transportation	6	-	-	5	-	-
Case 3	*Regulates himself when getting excited*	3	4	5	2	5	6
	Plays appropriately during his free time at home	3	3	4	2	3	4
	Expresses himself and gets adults’ approval before going somewhere outside	5	7	7	7	8	7
	* Goes out to join activities with other kids and has more interactions	2	4	3	6	6	7
	* Performs appropriate interaction behaviors when meeting people/children	6	8	7	2	8	8
	*Parent sets up daily routines between family and work to bring the child go out to park or do home training program* *s*	4	7	8	3	6	8
Case 4	Eats meals at home independently and keeps the body and table clean	4	-	-	2	-	-
	Puts on clothes independently	5	-	-	4	-	-
	Does and revises homework with concentration at home	5	-	-	3	-	-
	Engages in games by herself for 15 min at home	4	-	-	4	-	-
	* Performs appropriate social behaviors when playing with other kids at the playground or party in the community	3	-	-	4	-	-
	Brushes teeth routinely with adults’ assistance	3	-	-	4	-	-
	Controls emotion when things do not fall in with her wishes	3	-	-	5	-	-
Case 5	*Goes to bed by 9:30 p.m. and has the story time completed before that*	1	6	4	1	7	5
	Knows the name of tools and uses them in appropriate ways at home	3	5	5	3	6	5
	Tidies up personal belongings at home	7	7	6	5	7	6
	*Completes homework with motivation to learn the stroke sequence at home*	6	6	6	5	6	6
	Plays toys with his little sister	4	7	5	3	7	6
	*Interacts with other siblings during the reading time at home*	5	8	6	4	6	6
	* *Joins other kids’ plays by asking first at clubhouse or church*	1	4	5	2	4	5
	Parent incorporates school activities in the child’s learning activities at home	1	6	4	1	6	4
Case 6	*Does homework with concentration for 30 min at home*	2	6	2	1	6	1
	* Plays with other kids appropriately at playground or friends’ social events	5	5	6	2	6	5
	* Communicates with other kids or adults appropriately during play/daily life	3	8	7	2	9	5
	Pays attention to put on socks on his own	4	9	7	3	9	8
	Plays games and responds appropriately when losing the games in play	1	6	3	1	7	2

Italicized goals indicate that they were dealt in the OPC sessions. * indicates the goals related to children’s community participation. Abbreviation: FU, follow-up.

**Table 3:** Aggregated scores of outcome measures related to goals and community participation over time.

				Difference across Time		
Outcome Measures (Score Range)	Pre Mean (SD)	Post Mean (SD)	FU Mean (SD)	Pre vs. Post Mean (SD)	Post vs. FU Mean (SD)	Pre vs. FU Mean (SD)
COPM for all goals						
Child performance (range 0–10)	3.83 (0.85)	6.32 (0.66)	5.85 (1.21)	2.50 (0.95) *	−0.48 (1.13)	2.02 (0.42) *
Parents’ satisfaction (range 0–10)	3.55 (1.59)	6.58 (0.93)	5.73 (1.41)	3.03 (1.84) *	−0.85 (1.63)	2.18 (0.26) *
COPM for goals specific to community participation						
Child performance (range 0–10)	3.50 (1.73)	5.75 (1.19)	6.03 (1.27)	2.25 (0.65)	0.28 (0.98)	2.52 (1.23)
Parents’ satisfaction (range 0–10)	3.50 (1.91)	6.50 (1.68)	6.25 (1.44)	3.00 (1.78)	−0.25 (1.55)	2.75 (0.87)
YC-PEM						
Frequency (range 0–7)	3.30 (0.42)	3.20 (0.31)	2.25 (0.51)	−0.09 (0.31)	−0.95 (0.58) *	−1.05 (0.47) *
Involvement (range 1–5)	3.34 (0.45)	3.94 (0.51)	3.75 (0.48)	0.60 (0.49)	−0.19 (0.23)	0.40 (0.59)

* indicates the change scores beyond clinically important change of 2 points in parent-identified goal performance and satisfaction or beyond the minimal detectable change value of 0.7 points in children’s community participation frequency and involvement. Abbreviations: COPM, Canadian Occupational Performance Measure; YC-PEM, Young Children’s Participation and Environment Measure.

**Table 4:** Aggregated scores of outcome measures related to children’s HRQOL and parents’ mental health and parenting competence over time.

				Difference across Time		
Outcome Measures (Score Range)	Pre Mean (SD)	Post Mean (SD)	FU Mean (SD)	Pre vs. Post Mean (SD)	Post vs. FU Mean (SD)	Pre vs. FU Mean (SD)
Kiddy-KINDL (range 0–100)						
Total	57.03 (5.85)	60.42 (1.90)	55.47 (5.13)	3.39 (4.28)	−4.94 (3.93)	−1.56 (3.24)
Physical wellbeing	67.19 (5.98)	79.69 (5.98)	75.00 (8.84)	12.50 (5.10)	−4.69 (11.83)	7.81 (7.86)
Emotional wellbeing	65.63 (6.25)	64.38 (6.25)	63.75 (6.25)	−1.25 (0)	−0.63 (0)	−1.88 (0)
Self-esteem	54.69 (7.86)	51.56 (10.67)	53.13 (8.07)	−3.13 (3.61)	1.56 (9.38)	−1.56 (7.86)
Family	57.81 (9.37)	62.50 (5.10)	53.13 (16.54)	4.69 (10.67)	−9.38 (13.01)	−4.68 (16.44)
Social contacts	43.75 (10.21)	43.75 (11.41)	39.06 (5.98)	0 (16.93)	−4.69 (9.38)	−4.68 (13.86)
School functioning	53.13 (15.73)	57.81 (5.98)	51.56 (18.66)	4.69 (10.67)	−6.25 (15.31)	−1.56 (10.67)
DASS-21 (range 0–42)						
Stress	11.50 (8.39)	10.50 (7.19)	10.50 (4.12)	−1.00 (2.00)	0 (5.89)	1.00 (6.63)
Anxiety	3.50 (3.00)	2.50 (2.51)	3.00 (2.00)	−1.00 (2.00)	0.50 (1.00)	0.50 (2.51)
Depression	5.50 (5.00)	3.00 (2.58)	4.50 (1.91)	−2.50 (4.43)	1.50 (2.51)	1.00 (3.46)
PSOC						
Satisfaction (range 9–54)	30.00 (5.29)	30.75 (8.30)	28.50 (4.79)	0.75 (4.50)	−2.25 (5.80)	−1.50 (1.73)
Efficacy (range 7–42)	25.25 (6.18)	26.50 (2.88)	27.20 (3.77)	1.25 (4.03)	0.75 (6.02)	2.00 (7.83)
HCCQ (range 1–7)	5.70 (0.91)	6.43 (0.58)	-	0.73 (0.37)	-	-

Abbreviations: DASS-21, Depression, Anxiety and Stress Scale-21; PSOC, Parenting Sense of Competence Scale; HCCQ, Health Care Climate Questionnaire.

**Table 5:** Categories and sub-categories for parents’ experience of OPC.

Category	Sub-Categories with Examples
Increased insight and learning	Sub-category 1: New insight into child’s difficulties The parents understood which time slot in the day that the child had the best emotional status. Sub-category 2: New insight into parents’ needs The parents gained an insight into how they are supposed to train with the child properly. Sub-category 3: Learning new strategies, skills, or thinking models The parents learnt techniques that could be applied to see how time could be arranged for the child’s activities. The parents could think about what is the most ideal way to solve the child’s problem slowly.
Experiencing changes in their child	Sub-category 1: Increased participation in home activities The child completed homework within a reasonable time frame. The child read more stories and did housework together with the parents and siblings. Sub-category 2: Increased emotion or confidence The number of times the child lost their temper dropped. The child built confidence in school life.
Positive coach-parent relationship	Sub-category 1: Felt supported or encouraged The parents felt that the coach gave good advice. The parents were encouraged to keep working towards the target. Sub-category 2: Felt understood The parents felt that the coach understood their difficulties and the situation in Hong Kong.
Factors affecting coaching experience and suggestions	Sub-category 1: Disturbed by social issues or seasonal holidays Schools were closed owing to social unrest, and the child’s whole routine was messed up. Sub-category 2: Delivery mode and location of coaching Some parents preferred face-to-face coaching and some parents preferred internet-based coaching to show their home environment to the coach. Sub-category 3: Number of coaching sessions The parents wanted more coaching sessions to achieve their goals or build better habits to train their child. Sub-category 4: Frequency of coaching sessions The parents wanted more than one week to observe the child’s improvement or have more time to apply the strategies. Sub-category 5: Additional suggestions The parents suggested that the coach could provide access to a resource book, email/mobile message reminders, or parents’ education before or during OPC.

## Appendix A. Guiding Questions Used in Semi-Structured Interview of Parents, after OPC Intervention

1. What was your overall experience of the parent-coaching training?

Probe Q1: What have you learnt during the parent-coaching period?

Probe Q2: What did you like most (or least) during the parent-coaching period?

Probe Q3: During the parent-coaching period, what difficulties did you encounter?

Probe Q4: How closely did this parent-coaching meet your expectations?

Probe Q5: How would you describe your relationship with the coach?

2. How did the parent-coaching training help you or your child?

Probe Q1: How has the parent-coaching training helped your child to engage in home activities?

Probe Q2: How has the parent-coaching training helped your child to engage in community activities?

Probe Q3: How has the parent-coaching training helped to improve your child’s psychosocial health?

Probe Q4: How has the parent-coaching training affected your psychosocial health?

3. During the study period, how satisfied were you with the parenting-coaching process?

Probe Q1: What did you think about the coaching schedule? Or what did you think of the maximum number of sessions being eight? Or what did you think about the sessions being once a week?

Probe Q2: What did you think about each sessions being one hour long?

Probe Q3: What did you think about the delivery method (face-to-face or internet-based)? What did you think if we deliver the parent-coaching training through (internet-based or face-to-face)?

4. Would you recommend the parent-coaching training to other parents in need? If yes, how would you explain the intervention to them? (If no, why would you not recommend it?)
5. Lastly, what improvements to the parent-coaching training would you suggest, if it was to be applied in Hong Kong in the future?

## Appendix B. Narrative Description of OPC Sessions and Goal Achievement of Each Participant

1. Case 1 (a boy with autism and developmental delay, aged 5.25 years)

Both parents of Case 1 were coached for six sessions. During the first session, the parents considered that concentrating on completing homework within 30 min after school, while remaining emotionally stable, was the most important goal for their child. They reported many concerns regarding the child’s emotional stability, and the impact of his engagement in academic tasks. The OPC coach directed the parents’ focus to the days on which the child could complete the homework timely. During the solution-focused talk, the mother noted that the child might be more obedient if he was told about what to do one day before. The father also mentioned that the use of iPad/television as a reward after homework completion worked sometimes. The OPC coach instructed the parents to notice the timing and details around using those strategies in their daily routine, to which the parents agreed.

The second session was postponed for one week, due to school suspension as a result of the social unrest happening at the time. In the session, the parents reported an improvement in the child’s performance in completing homework, but they were unable to continue implementing the strategies during the school suspension period. The parents also had concerns about the child’s slow pace in writing Chinese words with complex strokes, and the over-use of iPad/television as the reward. The parents were encouraged to explore other strategies to write the complex words efficiently (e.g., breaking down the words or completing the same word in a row), and alternative rewards (e.g., allowing playtime with his brother) that could support the child’s performance.

In the third session, the parents reported further improvement in the child’s performance. They also identified the best timing for the child to engage in Chinese homework (e.g., good quality sleep the night before). Subsequently, a new goal emerged from the parents’ concern regarding the child’s emotion and disruptive behavior during piano lessons. After eliciting the parents’ knowledge, the mother decided to use a similar strategy, in other words, to let the child know what the piano teacher would teach in the next lesson. The mother would also practice with the child, letting him familiarize himself with the learning topics.

The fourth session was held two weeks later, as the mother had work commitments. In the session, the parents reported that the child became more cooperative and, in fact, the mother had not told him about the teaching content of the next lesson in advance. Instead, she had modified the strategy and asked the piano teacher to tell the child directly about what she would teach him at the beginning of the piano lesson. This modified strategy did enhance the child’s emotion and cooperation successfully.

During the remaining sessions (fourth, fifth, and sixth), the parents shifted their focus to another goal, which was that the child could complete homework during Christmas and Chinese New Year holidays. However, they struggled to identify successful strategies every time, because their holiday schedules were varied. The OPC coach shared several ideas (e.g., sorting out types of homework based on difficulty, offering breaks for lengthy homework), and invited the parents’ comments on the ideas. The parents decided to let the child complete difficult homework when he was emotionally stable, and shift to easy/interesting homework when he was tired. The plan was modified twice over the fifth and sixth sessions, when the child felt distressed and unsettled. For example, in the sixth session, the mother reported that the child suddenly stopped doing the homework after being asked to correct a wrong stroke sequence of a Chinese word. The OPC coach led the parents to think about what they observed at that moment, and how they could maintain the child’s motivation for homework completion. Additional strategies were further generated from the parents’ reflection; those were, to allow the child to make mistakes, but guide him to find and correct mistakes after the completion through game-playing approaches. These strategies were used depending on the child’s emotional status.

In the fifth session, the father reported an incident that had impacted on the child’s emotional state. The child had bit his brother’s arm when the brother did not want to share the new toy with him. The OPC coach facilitated the parents’ reflection on their understanding of their child’s behavior, and identification of possible strategies to prevent or deal with the behavior in the future (e.g., educating the child and reaching an agreement before playing with new toys, or using a timer to take turns to play). Similarly, in the sixth session, the mother reported an occasion where the child had suddenly started to cry and refused to eat lunch at home. Through the OPC coach’s guidance, the parents concluded that this incident might have been caused by the differences in the child’s routine, as they usually had lunch outside on the weekends. The mother further reported that she made use of the timer and gave the child time to calm himself down. Surprisingly, these strategies worked. The parents planned to continue using these strategies to help the child calm down when needed, as well as teach him to count down and take deep breaths as a self-soothing strategy.

In summary, three out of the seven goals were addressed in the six sessions (see Table 2). One goal was particularly related to the child’s community participation. Unfortunately, the remaining OPC sessions were terminated because of the outbreak of COVID-19, and the post-intervention measures were completed immediately. The parents also completed the follow-up measures two months after post-intervention.

2. Case 2 (a boy with autism, aged 4.00 years)

The mother of Case 2 was coached for one session only. During the session, it emerged that the mother’s priority was that her son followed her instructions during the morning routine, and left for school on time. The mother used a problem-oriented narrative to describe her son’s stubborn tendency, in other words, her son would cry and require time to calm down if things did not go the way he wanted. This would delay the arrival time at school in the morning. The OPC coach guided the mother to think about what happened in a good morning, and explore possible strategies. The mother reported that she used candy/seaweed as the reward to motivate her son, or used pictures to explain things along the road, which sometimes worked. Furthermore, if the iPad or iPhone were used as the reward, the child would comply. However, the mother hesitated to reward her son with the iPad/iPhone, as she was afraid that people would think that she was not a good mother. The OPC coach elicited her to reflect on the reasons behind this fear. The mother proposed to give her son the opportunity to watch the iPad or iPhone for ten minutes whenever they arrived at school on time (rather than along the way to school) as the reward. Unfortunately, the second session was postponed for one week because of the social unrest and, later, the child was ill and required hospitalization, resulting in the mother’s decision to withdraw from the research. Thus, no post-intervention or follow-up measures were completed.

3. Case 3 (a boy with autism and developmental delay, aged 5.50 years)

The mother of Case 3 was coached for the whole eight sessions. In the first session, the mother reported that the most important thing for her son was to have a regular morning routine, in order to get ready for school without any conflict. The OPC coach guided the mother to think about what she would like to happen in her son’s morning routine, and to explore strategies that might work. For example, her son was a visual learner, so the mother, as a casual architect, decided to use her skills to make a series of visual cards that could be shown to her son, letting him know what he needed to do in the morning. She also planned to use verbal prompts and reward, to encourage her son to complete the morning routine. The mother agreed to try the plan one morning in the following week. She would generate the visual cards, one day before executing the plan, and set the alarm in order to get up earlier.

In the second session, however, the mother reported that she only made some of the visual cards. Her son understood the steps of the cards, but was slow and felt unmotivated to complete the morning routine. He required a lot of verbal prompts from the mother, and ended up upset and crying. The OPC coach supported the mother and they worked together to explore alternative strategies (e.g., encouragement-oriented prompting, aiding in completing difficult steps, or using sensory play as the reward). However, the following week was affected by the social unrest, postponing the third session for one week.

During the third session, the mother reported that the plan had not been successfully implemented, as the school had re-opened for two days and their morning was very rushed. She also disclosed that she was tired from the previous night because she had spent time sorting her son’s toys and tidying up the home, and had gone to sleep late. This impacted on her energy level and she did not feel up to try the entire plan. Later she recognized the organization of her timetable as being important, in order for her to have sufficient energy. The mother identified that setting an alarm to go to bed by 12 a.m. could be a workable strategy, and she also planned to use a timer and visual cards to prompt her son to tidy up his toys after free play in the afternoon.

In the fourth session, the mother reported that she was unable to manage her time and went to bed by 12 a.m. Her sleep was also disrupted a few times by her second son at night. The original morning routine plan was missed out completely, and became less important for her. Instead she wanted to manage her time in order to organize the home (i.e., tidying up all her son’s toys and sorting them into lockers). Strategies were identified, through the OPC coach’s guiding questions, and included involving her son and husband in putting the toys away. At the same time, she put forward the idea of involving her husband in part of her son’s morning routine (i.e., taking charge in playing warm-up games or jumping on the trampoline with her son).

In the fifth session, the mother reported that she had had an unexpectedly busy week at work, and did only a little sorting and planning. Through guided reflection, the mother expressed that she had a perfectionist tendency, which made her want to sort out everything at once and, if not, she would feel that she could not do anything next. The OPC coach worked with her to narrow down the goal, and develop a step-by-step plan that began with two categories of toy sorting. Furthermore, the mother reported that her son had enjoyed playing matching games and trampoline with her husband in the morning, and she would continue with the plan. Meanwhile, her son had received a therapeutic listening program, requiring him to wear a special headphone for 30 min in the morning. The child seemed more calm and willing to follow instructions in the morning, according to the mother’s observation.

In the sixth session, the mother had managed to complete almost all of the domestic chores the night before. She planned to continue packing her son’s toys into the lockers, by narrowing the task down and labeling the items. When asked what the most important thing for her at that moment was, the mother returned to the previously unfinished goal regarding her son’s morning routine, and revealed that she wanted it to be extended to his after-school routine at home. The OPC coach guided her to think about what she wanted, and she created five-step morning and after-school routines for her son. The mother would use the visual cards to let her son know the steps and try out the routine plans for two days in the coming week. However, in the seventh session, the mother explained that the routine plans had not been implemented due to the busy holiday preparation. She had only been able to organize the house, but she felt comfortable with her time schedule and routine, and ready to implement the activities in the after-school routine with her son. In addition, while he still wore diapers at home, she had noticed that her son had started telling her when he needed the toilet. So, she created another goal for her child, which involved him going to the toilet every 45 min, without wearing diapers at home or when he expressed the need. Through collaborative performance analysis, she devised a reward system, to be trialed for half a day in the coming week, and an electronic alarm would be used as a reminder for her son to go to toilet regularly.

In the last session, the mother reported that she had modified the types of reward (originally collecting five points to get a chocolate croissant at the end) from small and instant ones (i.e., candy) to big and delayed ones (i.e., going to a theme park). The child was able to express the need to go to the toilet on two occasions. However, the mother did not use the system consistently, owing to the sickness of the child over the previous week. The OPC coach guided the mother to apply the successful reward system to other daily routines. The mother thought about behavior at the dinner table and homework compliance, and planned to extend the reward system to these areas.

In summary, two of the six goals were addressed in the eight sessions (see Table 2). None of the two goals were related to the child’s community participation. Since the number of sessions had reached the maximum, the OPC intervention was concluded. The mother completed the post-intervention and follow-up measures.

4. Case 4 (a girl with developmental delay, aged 5.25 years)

The mother of Case 4 was coached for one session only. During the initial stage of the session, the mother specified that she wanted her daughter to be more motivated to engage in academic-learning activities, for 30 min at home, during weekday evenings. The OPC coach used collaborative performance analysis to guide the mother to think about a preferred future and, through solution-focused conversation, to identify strategies that could support her daughter’s performance. Some strategies that were identified as being useful sometimes included having enough sleep the night before, incorporating motor activities that did not require sitting, using snacks as the reward after completion, or reducing the time of the activities. When the OPC coach moved it forward to action identification, the mother’s mobile phone had no battery charge, and the session ended abruptly. After the participant’s phone was recharged, she sent a text-message to the OPC coach, enquiring why she had been asked to identify solutions by herself, rather than receiving advice from the coach. As she preferred an expert-directed approach, she decided to withdraw from the study. No post-intervention or follow-up measures were completed.

5. Case 5 (a boy with developmental delay and dyslexia, aged 5.33 years)

The mother of Case 5 was coached for six sessions. In the first session, the mother identified the two most important things for her son. One was to complete the morning routine without bargaining behaviors, and the other was to complete the bed-time routine by 9 p.m. and go to bed by 9:30 p.m. For the morning routine, the mother was encouraged to identify a strategy that could motivate her three children (including the target child) to follow the rules. Strategies included processes around breakfast preparation, for example, asking the children what foods they would like to eat, preparing the necessary materials, and making breakfast in the morning. For the bed-time routine, the mother identified that watching television until bedtime could be a good motivator, provided that the children had showered, eaten, and completed their homework on time. The mother agreed to implement the two plans in the following week.

In the second session, the mother reported that the strategy for the morning routine had worked very well with her children, and they were now able to complete it smoothly. However, for the bed-time routine, watching television as a reward had only been successful on specific days, in other words, when the child did not receive intervention training and was back home at 5 p.m. When the child had to attend intervention after school he arrived home around 7 p.m., and by then was tired and just wanted to have free play. Furthermore, the child’s father usually came back home around that time, and had dinner together with the children. Before/after dinner, the children played with their father, or watched television, which delayed the bed-time routine. Through the mother’s reflection and knowledge elicited by the OPC coach, she put forward two strategies to be trialed in the coming week. One was to prepare different foods for her children (children’s favorite meals) and husband (ordinary meals), to incentivize the children to eat quicker. The other was to involve her husband in the bed-time routine by reading books to the children (instead of watching television) after dinner.

In the third session, the mother seemed frustrated as her son was still going to bed around 10 p.m. on the days when he had the intervention. The strategy of separate meals for her children and husband had been successful, but her husband did not feel comfortable reading books to the children. The mother reflected that it was understandable as her husband was not good at reading books and was also tired after work. The mother decided to create a schedule, and educate the children about the evening routine by using a whiteboard. In addition, the children were allowed to watch television if they completed the evening routine on time. The mother also relaxed the bedtime from 9:30 p.m. to 10 p.m., on those days the child came back late, to allow room for a buffer. In addition, during story time, the mother wanted to encourage her son to read more books and express his ideas, to reinforce his learning from school. She discovered who to approach at the school, in order to find out the school themes in advance, and where she could borrow similar books. She also planned to facilitate a story sharing time among her children during the weekends.

In the fourth session, the mother reported that her children did not get the meaning of the evening routine written on the whiteboard. Therefore, she had decided to put up pictures to assist their understanding of what they needed to follow in the evening routine for the next week’s plan. The mother was able to obtain the school theme from the teacher and incorporated it into the bedtime reading. Meanwhile, the mother reported that she had recently taken her child to the playground near the school, or to the clubhouse after school. Her son was able to invite his classmates or other kids to play together, by sharing snacks with them. Since this was one of the mother’s goals identified in the goal setting session, the OPC coach encouraged her to continue the initiative. For the remaining time, the mother expressed a need for her son to complete Chinese homework with a proper sitting posture at home. The OPC coach guided her to review her son’s current sitting posture and elicited her knowledge about the ideal one. Soon, the mother realized that the height of the chair was relatively lower than the table, so that her son had to lean his body toward the table, and sometimes the hand he used for writing was not placed on the table. The mother showed willingness to address this issue.

In the fifth session, the mother reported that she had managed to get her children to go to bed around 9:30 p.m. by following the previously discussed strategies on the days her son had the intervention. The child started to express his ideas, and interacted with his little sister during the story-time. The mother also went to the library to borrow books with similar themes to the ones being learned at school, to reinforce learning. Additionally, it was unexpected by the OPC coach that the mother asked her husband to share her workload and help with the child’s homework. Since her husband appeared to accept this duty, the mother would continue it. The mother also reported that the chair, after the height adjustment, improved the child’s sitting posture and performance in writing Chinese. She also adjusted the height of the chairs for her other two children. For the remaining time, the mother raised a concern about her son’s poor performance in using scissors to cut lines/shapes accurately for homework. Several strategies were generated to enhance his performance, for example, to widen the lines by using color markers to easily trace lines while cutting, and to place direction signs in each turn to remind her son to turn the paper while cutting. The mother planned to apply these strategies while sharing a fun project with the child, such as making a Chinese New Year card.

In the sixth session, the mother reported that the child was able to accurately cut the straight lines that were highlighted in red using the marker, and to turn the paper using the non-dominant hand while cutting. However, he needed constant reminders to place his elbow on the table and maintain good sitting posture. He also did not enjoy making the card and stopped it after cutting six pieces. After the OPC coach-guided reflection, the mother realized that the paper for making the cards seemed too thick for the child to cut. Thus, she planned to organize a Chinese lantern making activity in the coming weeks, as this was usually a homework task for the Lantern Festival. The mother would prepare different sets of materials for the child and his older sister, divide the task into smaller portions, and complete some portions daily. Highlighting with color markers and reminders for the posture were continued, and the mother also thought that cutting straight (not curve) lines was most suitable for her son’s ability at that moment.

Unfortunately, the remaining OPC sessions were terminated because of the same reason (i.e., the impact of COVID-19) mentioned previously for Case 1. In summary, four of the eight goals were addressed in the six sessions (see Table 2). One goal that was dealt with was related to the child’s community participation. The mother completed the post-intervention measures immediately and the follow-up measures two months later.

6. Case 6 (a boy with autism and developmental delay, aged 5.25 years)

The father of Case 6 was coached for three sessions. In the first session, he considered his son’s completion of homework within one hour to be the most important goal to achieve. The father reported that the child was constantly asking for assistance or refusing to do the homework, especially when the subject was Chinese. After prompting for the father’s reflection, he reasoned that his son might not know how to write Chinese words, particularly within the grids. Since the child’s Chinese homework was supervised by his wife, the father agreed to invite his wife to join the next OPC session. In addition, the father identified several strategies that he planned to try out for enhancing his son’s compliance with homework completion. These included watching television as the reward, and physical demonstration of how to write simple Chinese words (within 4 strokes).

Both mother and father attended the second session. The father reported that his son had shown some improvement, after he had been instructed on how to break down the Chinese for writing, and was also more willing to complete the homework that contained less complicated Chinese words on his own. However, the mother reported that the child was still unable to write Chinese words with complicated structures, even though she had taught him the stroke sequence twice. Through the OPC coach’s guidance, the mother reflected that she, at times would get very angry if the stroke sequence made by the child was wrong, and would require 5–10 min to calm herself down. In the meantime, the child would be offered a break to watch television before continuing the homework. The parents identified the inconsistency in their parenting styles, especially regarding the use of television as a reward, in other words, at the end (father’s style), or as a break in-between (mother’s style). The break was important for the mother, as it helped her to calm down, however she agreed to use it as little as possible. The OPC coach shared his view about breaking down complicated Chinese words into small parts, given that the child was able to write simple Chinese words. The mother considered it as a possible strategy, and agreed to try it out. Regarding the reward for homework completion, after being prompted for an alternative, the father suggested taking his son out to the playground.

In the third session, the mother was not available. The father reported that the plan for taking the child to the playground as the reward had worked only for school days but not for holidays. He reasoned that there was not much homework over Chinese New Year holidays, and his son wanted to watch television as the reward. He would continue the playground plan after the holiday period. He also did not know whether breaking down Chinese words supported the child’s performance. This plan would be reviewed when his wife attended the session later. For the remaining time, the father prioritized that he wanted his son to play with other children with no fighting, and demonstrate appropriate behaviors when visiting friends or engaging in the community. He noticed that the child behaved differently (i.e., more uncooperative) when he was present, compared to when his wife was present (more obedient). Through guided reflection, the father mentioned that he usually acted as a friend of his son, which could explain the difference in behavior. He felt it was necessary for him to change this, and to show his son that he would not be manipulated easily. The father proposed a punishment system, which involved reducing the time spent watching television if the child had a fight with other children, as well as removing his son from the conflict situation immediately to allow him to calm down. Afterward, he would listen to his son and educate him about appropriate behavior when playing with other children.

Owing to the impact of COVID-19 starting in March 2020, the father agreed to stop the OPC intervention earlier. In summary, two out of the five goals were addressed in the three sessions (see Table 2). One of the goals that was addressed referred to the child’s community participation. The father completed the post-intervention measures immediately and the follow-up measures two months later.

## Appendix C. Illustrative Quotations of Each Identified Sub-Category for Parents’ Experience of OPC

**Table A1:** Quotations of Identified Sub-Categories for Parents’ Experience of OPC

Category and Sub-Categories	Quotations
Increased insight and learning	
Sub-category 1: New insight on child’s difficulties	Need to know, need us more to understand which time slot in a day that he (the child) has the best emotional status. Then I will make use of that time, enabling him to complete the things that I want him to do. (Case 1′s mother) I never been that kind of coaching. Sometimes it is hard for parents to see the blind spot, how we interact with our kids, or how we teach our kids. We just use the way how we learnt, and then teach the kid. Maybe my son is not learning with the same method as me. (Case 5′s mother) By taking these classes, it does give me more patience and understanding of my son’s problems. (Case 6′s father)
Sub-category 2: New insight on parents’ needs	I guess it (what I like most during the coaching period) is the space, I don’t feel so pressured which I feel more comfortable in terms of doing it but I, like again, it’s really depends on the self-discipline. So it’s, it’s good that I have a coach. (Case 3′s mother) Give me an insight of, you know, how you suppose to train properly with the kid. In fact, the things we actually give a lot of rewards on TV time, and sometimes, me and my wife is (not consistent), because I have my style of teaching kids, and my wife has another style of teaching the kid. And that’s our problem. Because we won’t be consistent. (Case 6′s father)
Sub-category 3: Learning new strategies, skills, or thinking models	I learnt some techniques, those are, he (the coach) shared some treasured experience that we could try to apply to see how much the child could improve or how we arrange time (for the child’s activities). Overall, it helps the parents and the child. (Case 1′s father) I learnt to look at, I think I learnt some sort of thinking model that, if I hit a problem, I would think what is the most ideal way that I wanted. And I try to think from that angle, and do it slowly … Like what would be ideal, and how do I achieve it. And then, and then, I also learnt to start small, start slow. (Case 3′s mother) I remember there were occasions I failed. The first one was … The second one was making an environmental-friendly lantern my son would bring it to school. I planned to do it with my son during the 6th meeting. I did not make good use of the holiday and failed. Even, I failed to make the lantern with my son. I learnt skills from the coaching sessions. (Case 5′s mother)
Experiencing changes in their child	
Sub-category 1: Increased participation in home activities	Maybe for doing the homework. He (the coach) told us how to do to make the child feel interested to do homework. Using different techniques to communicate with him (the child), I think this aspect (doing homework) improved. (Case 1′s mother) My son talks with us more and he plays less by himself. Before joining the parent coaching, if we don’t stop him, he will keep playing the train with himself for more than 1 h. After the coaching, we start to interrupt him and invite him to play with others … We read more stories together and do the housework together, from 0 to once or twice a week. (Case 5′s mother) It (coaching) helps a little bit with writing, and helps a little bit with putting on the socks. (Case 6′s father)
Sub-category 2: Increased emotion or confidence	Even in the interest class he (the child) takes, the teacher also faced the situation where the child has a bad mood. When not good, he (the coach) told us that, actually, we could tell the teacher directly and ask her to give advanced announcement (about what she would teach) … improved, improved a lot actually for the emotion … that is he (the coach) had taught us some techniques and we tried how to communicate with our child to control his emotion. That is the emotional responses at home, and the number of losing his temper was dropped. (Case 1′s mother) My son is very shy and afraid to express his feelings. He does not know how to ask help or raise questions … I let him practice by staying behind after school and enforce his learning in our conversation. He (the coach) provided a lot of suggestions and possibilities to help my son to build confidence in his school life. (Case 5′s mother)
Positive coach-parent relationship	
Sub-category 1: Felt supported or encouraged	I feel like (the coach is) a very experienced person who is very willing to share his experience, so as to let us know how to consider in every aspect, or in the aspect of arranging time, difficulty of challenge (of tasks), etc. That means, giving us a lot of treasured experience. (Case 1′s father) I’ m happy not because of the process of the coaching but it’s because of everything else, like because of the talking, because of the sharing session, and maybe the guiding of my own thinking process. So he (the coach) gives guidance and he also gives really good advice. (Case 3′s mother) First, being encouraged is most important. Second one is receiving very detailed suggestions that are very practical. As I have 3 kids, the time constraint is bigger for me, it is harder for me to take care them at the same time. I need detailed suggestions to execute my plan smoothly. He (the coach) had been encouraging me to keep going to my target. (Case 5′s mother)
Sub-category 2: Felt understood	Because he (the coach) is very professional. He understands the difficulties of parents. And he understands the situation in Hong Kong. (Case 5′s mother) He (the coach) is funny, he is willing to teach, and you know, I think we have a good relationship, understanding of, you know, his techniques and he understands mine, you know, situation. He is really listening. (Case 6′s father)
Factors affecting coaching experience and suggestions	
Sub-category 1: Disturbed by social issues or seasonal holidays	I think, (it) is to do with the whole situation. It was first school holidays, a lot of, yes, so it’s just because of the social situation that schools stop. And because I have 2 kids at home, and when they don’t go to school, it’s, the whole routine messed up. And I’m at the moment of building my routine. And if it got messed up, it’s adding difficulties to build things. (Case 3′s mother) Um, yes, the holidays didn’t work as well. Because a lot of training require, you know, like, the repetition but let’s say, during Christmas holidays, we suppose to train him repetition, but a lot of time we have to go to other peers, other parties, and you know, when we go to the parties, you cannot, you cannot train him as well as at home, because there’s no more writing, there’s no more guidance, there’s no more rules. You know, everything went out the door, will be training. (Case 6′s father)
Sub-category 2: Delivery mode and location of coaching	I think that both have their advantages. Because, for internet, I can arrange the time. Going to the university takes us a few hours for return, just only for the transportation. If conducted through internet, it saves time. However, for face-to-face, we think there is a need to take the child to visit the coach at the first session, and so let him (the coach) observe the child’s conditions … Maybe, when there is chance in the future, maybe half-half, that is, half for the training conducted through face-to-face and half through internet. (Case 1′s father) It’s fine for me. Like meeting in person would be good, but, I don’t see there’s any difference if I have to do it on internet … Because, while I was at home, I was able to show my home environment to the coach, and he’s able to see something that I’ve done over the past week. So in that regard, online meeting is better. (Case 3′s mother) The face-to-face method is very useful. He (the coach) and my family live in the same district … I am so glad he does not mind coming to my home … I think it would still be good enough now. The coronavirus stopped us from meeting. It would be better to have face-to-face coaching at the beginning. After building trust and understanding the concept, we would move to internet-based methods such as Zoom. (Case 5′s mother) What do I like least? … Maybe the training area, because the university was, you know, disrupted. We have to do everything in the car. So maybe that I like least, but, you know, that is the problem of it … Face-to-face is actually better than anything else. (Case 6′s father)
Sub-category 3: Number of coaching sessions	I will definitely want more (sessions) because, like I said before, I feel it’s going slightly slow … I always refer it as a snowball. So I think that everything to begin with is slow … So if you have to build something, the foundation is always taking longer. So, I think, for anything to get built up or achieve, or snowballing, and, this time so far isn’t quite enough to make a base. So I think it needs, it needs longer. (Case 3′s mother) With longer coaching time, I will build better habits to train my son. It would be much easier for parents to enforce what we had learnt if there are 10 coaching sessions. (Case 5′s mother)
Sub-category 4: Frequency of coaching sessions	Maybe one to two weeks will be better for observing his improvement. It is because sometimes there are holidays, school suspension, maybe, slightly extending the frequency of the training during these periods. (Case 1′s father) I think maybe twice every 3 weeks, maybe more ideal for me. (Case 3′s mother) If possible, it would be better to meet every 2 weeks in the first and second period of the coaching. It would allow me to have more time to apply what he (the coach) is coaching. I mean the duration … My son will have more time to do the preparation. (Case 5′s mother)
Sub-category 5: Additional suggestions	Designing a handbook about “the most common 100 problems and solutions for coping with the difficulties faced by children”. In addition to every meeting, we can have this handbook and refer to it, to understand the guidance of using the techniques, and so let us to make the reference, to practice, to see whether it (the technique) can help the child. (Case 1′s father) Receiving an email or WhatsApp message between 2 weeks gap will be more helpful for the parent. The reminder would refresh key points which were discussed with the coach. (Case 5′s mother) I think my recommendation is to train the parents first, with a class of 2, and then, be go on, on the, focus on the kids instead. (Case 6′s father)
